# E5 treatment showing improved health‐span and lifespan in old Sprague Dawley rats

**DOI:** 10.1111/acel.14335

**Published:** 2024-09-19

**Authors:** Kavita Singh, Shraddha I. Khairnar, Akshay Sanghavi, Tanuja T. Yadav, Neha Gupta, Jay Arora, Harold L. Katcher

**Affiliations:** ^1^ Shobhaben Pratapbhai Patel School of Pharmacy & Technology Management SVKM's NMIMS Mumbai India; ^2^ Yuvan Research Pvt Ltd Mountain View CA USA

**Keywords:** cytokines, E5, health‐span, lifespan, longevity, rejuvenation

## Abstract

Aging and, in particular, the emergence of age‐related disorders is associated with tissue dysfunction and macromolecular damage, some of which can be attributable to accumulated oxidative damage. In the current study, we determine the potential of ‘plasma‐derived fraction (E5)’ for cellular rejuvenation and extending the lifespan of Sprague Dawley (SD) rats. This is a unique study wherein we have used 24‐month‐old rats and monitored them until the end of their lifespan with and without E5 treatment. In the present investigation, the SD rats were separated into two groups old control group and the treatment group (*n* = 8). The treatment group received four injections of E5 every alternate day for 8 days, and eight injections every alternate day for 16 days. Body weight, grip strength, cytokines, and biochemical markers were measured for more than 400 days of the study. Clinical observation, necropsy, and histology were performed. The E5 treatment exhibited great potential by showing significantly improved grip strength, remarkably decreased pro‐inflammatory markers of chronic inflammation and oxidative stress, as well as biomarkers for vital organs (BUN, SGPT, SGOT, and triglycerides), and increased anti‐oxidant levels. Clinical examinations, necropsies, and histopathology revealed that the animals treated with the E5 had normal cellular structure and architecture. In conclusion, this unique ‘plasma‐derived exosome’ treatment (E5) alone is adequate to improve the health‐span and extend the lifespan of the old SD rats significantly.

## Graphical abstract

In the present investigation, plasma derived E5 was evaluated for anti‐aging effect on old SD rats. This treatment exhibited great potential by showing significantly improved grip strength, remarkably decreased pro‐inflammatory markers of chronic inflammation and oxidative stress, as well as biomarkers for vital organs (BUN, SGPT, SGOT, and triglycerides), and increased anti‐oxidant levels. In conclusion, this unique ‘plasma‐derived exosome’ treatment (E5) alone is adequate to improve the health‐span and extend the lifespan of the old SD rats significantly.

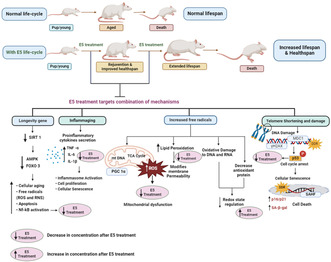

## 1 INTRODUCTION

Aging is the accumulation of various harmful changes that occur in the cells and tissues and are responsible for the elevated risk of illness and death (Chaudhary et al., 2023). Age is the single largest risk factor for a wide range of illnesses, including dementia, cancer, arthritis, and heart disease (Khandelwal & Gupta, 2023).

Modern gerontology places a high focus on the quest for safe, efficient ways to postpone or prevent the onset of aging‐related disorders, slow down or reverse the aging process, and lengthen lifespan (Cocco & Pandolfi, 2023; Tosato et al., 2007). Effective therapies that increase a person's healthy lifespan may have a major positive impact on productivity, healthcare costs, and quality of life (Kaeberlein et al., 2015). Bioactive peptides are a group of peptides having less than 50 amino acid residues that have function in living organisms or cell and have showed an anti‐aging efficacy that has attracted the scientific fraternity recently (Akbarian et al., 2022). It has been found that endogenous peptide formulations derived from the pineal and thymus glands possess anti‐oxidant, anti‐carcinogenic, and immunomodulating properties, which contribute to the extension of lifespan in rats, mice, and humans (Alamdari et al., 2021). Even though fruit flies lack pineal or thymus glands, similar beneficial effects have been observed in them (Alamdari et al., 2021; Anisimov, 2001).

The development of medications that can both lengthen health‐span (concept that emphasizes importance of living healthy and productive life) and lifespan (emphasizes on living a long life) has been the subject of several investigations (Barzilai, 2020). Recent studies demonstrated that it is possible to postpone and even reverse the biological aging process in hopes of minimizing the effects of age‐related disorders, which would be extremely beneficial for society and people's quality of life (Tosato et al., 2007). Studies have also shown that applying young blood plasma to various tissues and organs can reverse the consequences of aging. Blood plasma clinics are now being developed, and research is still being done to uncover all the health benefits of using young blood plasma (Bieri et al., 2023). Plasma derived exosome can be used to rejuvenate and increase the lifespan of SD rats.

In recent years, many scientists have worked on rapamycin, as an option for anti‐aging therapy. Rapamycin is an inhibitor of mTOR (mammalian target of rapamycin) that is a major regulator of the aging process. It has been demonstrated to lengthen life in several animals and delay or reverse multiple age‐associated phenotypes in mice including cognitive decline, reduce cardiac problems, and cancer cure (Anisimov et al., 2011; Dai et al., 2014; Halloran et al., 2012; Johnson et al., 2013). Despite these remarkable outcomes, adverse effects may restrict the ability of rapamycin or other mTOR inhibitors to prevent aging. The negative side effects include mouth ulcers, edema, higher circulating triglycerides (TG), impaired glucose homeostasis, poor wound healing, and gastrointestinal discomfort (De Oliveira et al., 2011).

According to studies utilizing young plasma, the acellular component of blood can be reversed in older animals. Fascinatingly, despite the blood–brain barrier, this functional improvement is seen in the brain as an improvement in memory and functioning (Ceylani & Teker, 2022; Kheifets & Braithwaite, 2019; Villeda et al., 2014). Exosomes are a class of cell‐derived extracellular vesicles of endosomal origin and are typically 30–150 nm in diameter (Di Bella, 2022). The plasma‐derived exosomes, a unique product can be used to improve the lifespan of Sprague Dawley (SD) rats. These exosomes are vesicles involved in intercellular communication and have been investigated for their biological roles and possible use as a disease biomarker and drug carriers. Exosomes with diameters ranging from 50 to 150 nm differ from microvesicles and apoptotic bodies that originate from the cell membrane of the parent cell. They show a diverse mixture of proteins, lipids, and nucleic acids depending on their cell origin. Various functional roles have been proposed, encompassing functions such as cellular waste vesicles, immunological roles, and facilitation of cell‐to‐cell communication. Moreover, recent research has explored the potential of exosomes as disease biomarkers (Jakubec et al., 2020; Skotland et al., 2017).

The current study is based on the therapeutic potential of young porcine blood plasma‐derived exosomes in a preclinical setting, emphasizing outcomes related to cellular rejuvenation and metabolic function. In continuation with our earlier reported study (Horvath et al., 2024), we hypothesized that the E5 a ‘plasma‐derived exosome’ that is the unique product of a unique process can be used to rejuvenate and increase the lifespan of SD rats (Horvath et al., 2024). Here, we employed size exclusion columns to separate vesicles of exosome size (EVs) derived from porcine plasma. The purity and anticipated properties of these vesicles were confirmed through transmission electron microscopy (TEM) and western blotting.

Our findings suggest that these plasma‐derived exosomes may hold promise as a potential therapeutic intervention for age‐related dysfunction of all organs assessed (Rosell‐Cardona et al., 2021).

## 2 MATERIAL METHODS

### 2.1 Chemicals and kits

Thiobarbituric acid and 5, 5′‐dithiobis (2‐nitrobenzoic acid) (DTNB) were procured from Sigma Aldrich, USA. Polyethylene glycol 6000, sodium chloride, sodium carbonate, triton X, and trichloro acetic acid were procured from Sisco Research Laboratories, India. Alanine aminotransferase (ALT), aspartate transaminase (AST), creatinine, blood urea nitrogen (BUN), and TG diagnostic kits were purchased from Transasia Biomedicals Ltd., India. ELISA kits like TNF‐α, IL‐6, p53, and NF‐κB were procured from Thermo Fisher Scientific, USA. CD9, CD63, and CD81 primary antibodies and goat anti‐rabbit IgG secondary antibody were procured from ABclonal Technology, USA.

### 2.2 Animals and treatment

24‐month‐old SD rats (Female, 300–350 gm) (Malik et al., 2024) were procured from the VAB biosciences, Hyderabad, India, and maintained at a facility for animals with a 12‐h light/dark cycle. Purified water and a multi‐nutritional pellet food (Nutrimix Laboratory Animal Feed, India) were accessible. The protocol was approved by the Institutional Animal Ethics Committee (IAEC).

The rats were randomly assigned to two groups: 1. Old control group (*n* = 8) and 2. Treatment group (*n* = 8).

### 2.3 Isolation of exosomes from porcine plasma

The platelet‐free porcine plasma was obtained from young animals (less than 1 year old), and its volume was measured. To this platelet‐free plasma, an equal volume of PEG solution in 0.5 M NaCl was added, and the combined mixture was incubated for 8–12 h at 4°C. After the stipulated time, the solution was centrifuged at 1000Xg for 5 min at 4°C, and the supernatant was discarded. The sediment collected at the bottom of the tubes was transferred into a vessel and mixed with enough physiological saline buffer to make a suspension. The suspension obtained in the above step was subjected to size‐exclusion chromatography on Sephadex G − 100. The fractions collected from size exclusion chromatography were recombined and concentrated. The concentrate was then mixed with sterile physiological saline solution and injected into rats through the tail vein.

## 3 CHARACTERIZATION OF EXOSOME

### 3.1 TEM

Purified exosomes were fixed with 2% paraformaldehyde. A 10 μL drop of the suspension was loaded onto a formvar coated grid, negatively stained with 2% aqueous uranyl acetate for 2 min, and examined under a transmission electron microscope Tecnai‐12 G2 using a digital camera.

### 3.2 Particle size and zeta potential determination

The mean particle size and zeta potential of the plasma derived fractions (E5) was measured by using the Malvern Zetasizer (Nano ZS, USA). For the determination, plasma fraction sample was dispersed in phosphate buffer saline; the suspension was sonicated so as to break down any aggregate formed during separation by centrifugation. One millilitre of this dispersion was filled with tilted position inside electrophoretic cell with the help of micropipette, caps were positioned at both the ends and inverted to check the presence of air bubble and the zeta potential was measured.

### 3.3 Nanoparticle tracking analysis

The size and concentration of plasma derived fractions (E5) were measured by using NanoSight NS300 (Malvern Panalytical, Malvern, UK) under the scattered setting. Each sample was loaded by syringe pump into the machine and five 10‐s videos were generated. For nano particle tracking analysis, sample was diluted further (1000 times) with water to get desired concentration. The entire process of sample measurement took about 15 min. This analysis was performed at Aimil Pvt. Ltd., Delhi.

### 3.4 Proteomic analysis

The changes from porcine plasma to E5 fraction at the proteome level was assessed. Proteomic analysis was carried out by using Orbitrap‐High‐Resolution Liquid Chromatography Mass Spectrometry (O‐HRLCMS) at SAIF facility, IIT Bombay. The instrument used was Q‐Exactive Plus Biopharma (Thermo Scientific) and the data was acquired with Thermo Scientific Xcalibur, Version 4.2.28.14. The specifications of the analytical column used was PepMap RSLC C18 2um, 100A x 50 cm. Mobile phase used was in the proportion of 85:15 (ACN: 0.1% Formic Acid in milliQ water). For analysis, the software—Thermo Proteome Discoverer 2.2 was employed. The database used was uniprotkb_pig_2024_07_10.fasta. With porcine plasma set as control the proteomic changes in E5 fraction was compared.

### 3.5 Western blotting

Expression of specific markers CD9, CD63, and CD81 in the sample of plasma‐derived exosome was studied using CD9, CD63, and CD81 (ABclonal Technology, USA) primary antibody and peroxidase‐labelled goat anti‐rabbit IgG secondary antibody. Radio‐immuno‐precipitation assay buffer (RIPA Lysis and Extraction buffer, ThermoFisher Scientific, USA) and protease inhibitor cocktail (Roche Diagnostics, USA) were used to extract the proteins from the plasma‐derived exosome samples. Protein estimation was done using Bradford reagent (Sigma‐Aldrich) at 595 nm using a UV spectrophotometer (Shimadzu UV‐1800) with UV WINLAB software. Samples were loaded at concentration of 30 μg/well and separated using 8% SDS‐PAGE. Immun‐Blot® PVDF membrane (Bio‐Rad, USA) using the Trans‐Blot® SD semi‐dry transfer cell (Bio‐Rad, USA). After 2 hr blocking at room temperature with 5% Non‐fat dried milk (NFDM) in 1X PBS, the membrane was exposed to respective primary antibodies in 5% NFDM overnight at 4°C. After washing with PBST (0.1% Tween‐20 in 1X PBS), the membrane was labelled with secondary antibody for 1.5 h at room temperature followed by PBST washes. The primary and secondary antibody as follows: CD9 (rabbit; 1:1000) (AB clonal Technology, USA), CD63 and CD81 (rabbit, 1:500) (AB clonal Technology, USA), and secondary antibodies (HRP conjugated goat anti‐rabbit IgG: 1:2000) (AB clonal Technology, USA). The blots were visualized using Bio‐Rad Molecular Imager® ChemiDoc XRS+ System with Image Lab™ Software with the help of chemiluminescence (ECL) reagent (Clarity Western ECL, Bio‐Rad, USA). Densitometry analysis was performed using ImageJ software.

## 4 EXPERIMENTAL PROCEDURE

### 4.1 Study design

24‐month‐old Female SD rats (300–350 g) were chosen for the experiment because majority of the preclinical studies in worldwide research are undertaken in males hence it was important to understand the effect of E5 treatment in female, female aging and female lifespan. For evaluation, repeated blood withdrawals and injections were needed and as the rats handle this better than mice hence, specifically rats were used for this study.

The effect of E5 treatment derived from porcine plasma is the basis of our fundamental discovery (Horvath et al., 2024).

The doses were calculated based on the weight of the animal and were administered intravenously to the animals of the treated group; a single rejuvenation treatment consisted of four injections given every alternate day for 8 days, the first dose started on the first day of the study. The same treatment was given to the rats of the experimental group from the 68th day following the first injection of E5, the third, fourth and last fifth treatment dosing was started at 181st, 280th, and 377th day of the study. A similar amount of sterile saline solution (placebo) was administered to the animals of the old control group as per the body weight of the rats. The body weight of the animals was monitored at the following time points: 0, 15, 30, 60, 66, 75, 86, 90, 105, 120, 135, 150, 165, 180, 195, 210, 225, 240, 255, 270, 285, 294, 315, 330, 345, 360, 375, 390, 405, 420 days. Blood samples were withdrawn from the retro‐orbital plexus at predetermined time intervals (0, 15, 30, 60, 66, 74, 81, 84, 86, 180, 195, 210, 279, 287, 294, 315, 376, 391, and 420 days) to determine the levels of inflammatory cytokines and other biochemical parameters.

Serum and plasma were separated from the blood; samples of each animal were evaluated for biochemical parameters. Plasma was separated by centrifugation from the blood samples of each animal and was used for evaluation of inflammatory markers that is, TNF‐α and IL‐6 as well as concentrations of p53 and NF‐kβ were determined in blood plasma. After the death of animals, the vital organs (brain, heart, lung, liver, spleen, kidney, uterus, and ovary) were harvested for testing of oxidative stress biomarkers (GSH, SOD, Catalase, and MDA) and histopathological studies.

## 5 END‐POINT EVALUATIONS

### 5.1 Body weight

The body weights of rats were recorded before the initiation of the treatment protocol and then after 15, 30, 60, 66, 75, 86, 90, 105, 120, 135, 150, 165, 180, 195, 210, 225, 240, 255, 270, 285, 294, 315, 330, 345, 360, 375, 390, 405, 420 days.

### 5.2 Grip strength

A grip strength meter was used to measure forelimb grip strength which represents the muscle strength of animals. The maximal pull force was briefly captured on a digital force transducer as the rat grabbed the meter's bar. Maximum tension was recorded as the rat was pulled away from the bar before the rat was let go. The time at which the rat released its forepaws from the bar was noted. At each time point, three successive measurements were made at 1 min intervals.

### 5.3 Biochemical evaluations

Blood samples were collected from the retro‐orbital plexus using heparinized capillary tubes before the treatment and on the 420th day of the experiment. One portion of the blood was kept in plain bottles from which serum was collected and stored for biochemical analysis. Further, the levels of serum glutamic‐pyruvic transaminase (SGPT) and serum glutamic‐oxaloacetic transaminase (SGOT) were carried out by the kinetic method recommended by the International Federation of Clinical Chemistry (IFCC). A high level of SGOT released into the blood may be a sign of liver or heart damage, cancer, or other diseases, while high levels of SPGT indicate liver damage. All the tests were performed with commercially available diagnostic kits on an Erba Mannheim biochemistry semi‐auto analyzer (Erba Mannheim, Germany).

Kidney function tests such as the determination of creatinine (mg/dL) and BUN (mg/dL) levels were measured. Also, blood TG levels were determined according to commercially available diagnostic kits on semi‐auto analyzer.

### 5.4 Oxidative stress evaluation

To evaluate oxidative stress parameters, the brain, heart, lungs, and liver were isolated after the experiment. A 10% tissue homogenate was then prepared in ice‐cold 50 mM PBS (pH 7.4) using a homogenizer and sonicated for 5 min. The resulting homogenate was centrifuged at 2000 g for 20 min at 4°C, and the supernatant was collected and stored at −20°C until further evaluation.

### 5.5 Estimation of malondialdehyde (MDA)

To estimate the extent of lipid peroxidation (LPO) in tissue homogenate samples of the brain, heart, lung, and liver, a method was employed using MDA as a marker. The samples were treated with a 1% phosphoric acid solution and an aqueous solution of 0.6% thiobarbituric acid. The resulting reaction mixture was then subjected to heating at 80°C for 45 min followed by cooling in an ice bath. Subsequently, it was extracted with 4.0 mL of n‐butanol, and the n‐butanol layer was separated. To determine the extent of LPO, the absorbance of the pink‐coloured complex formed was measured at 532 nm (Ohkawa, Ohishi, & Yagi, 1979).

### 5.6 Estimation of reduced glutathione (GSH)

The levels of GSH were measured in the brain, heart, lung, and liver tissue homogenate using the DTNB method, which involves treating 20 μL of the homogenate with 180 μL of 1 mM DTNB solution at room temperature. The resulting yellow color was measured at 412 nm using a microplate spectrophotometer (PowerwaveXS, Biotek, USA).

### 5.7 Determination of the catalase activity

The brain, heart, lung, and liver tissue homogenate (20 μL) were added to 1 mL of 10 mM H_2_O_2_ solution in the quartz cuvette. The reduction in optical density of this mixture was measured by using a spectrophotometer in UV mode at 240 nm. The rate of decrease in the optical density across 3 min from the addition of brain, heart, lung, and liver homogenate was taken as an indicator of the catalase activity present in the homogenate (Lück, 1965).

### 5.8 Estimation of superoxide dismutase (SOD) activity

The brain, heart, lung, and liver tissue homogenate (20 μL) were added to a mixture of 20 μL of 500 mM of Na_2_CO_3_, 2 mL of 0.3% Triton X‐100, 20 μL of 1.0 mM of EDTA, 5 mL of 10 mM of hydroxylamine and 178 mL of distilled water. To this mixture, 20 μL of 240 μM of nitroblue tratrazolium (NBT) was added. The optical density of this mixture was measured at 560 nm in kinetic mode for 3 min at one‐minute intervals. The rate increase in the optical density was determined as an indicator of the SOD activity (Paoletti & Mocali, 1990).

### 5.9 Pro‐inflammatory cytokines (IL‐6 and TNF‐α) and p53, NF‐κB

The cytokines and p53, NF‐κB were estimated in plasma, which was separated from the blood of animals and kept at −20°C until the execution of the assay. The pro‐inflammatory cytokine levels including TNF‐α and IL‐6, p53, and NF‐κB were determined by using a sandwich ELISA kit (Thermo Fisher Scientific, USA), according to the manufacturer's protocol, and the values were calculated from the optical density.

### 5.10 Histopathology of vital organs

Brain, heart, spleen, kidney, lung, liver, uterus, and ovary tissues fixed in neutral buffered 10% formalin solution were embedded in paraffin, and serial sections (3 μm thick) were cut using a microtome (Leica RM 2125, Germany). The representative sections were stained with hematoxylin and eosin and examined under a light microscope (Leica, Germany). The histopathological data was objective and the sections were screened by a pathologist blinded to the treatments.

### 5.11 Statistical analysis

Data were expressed as mean ± standard error of the mean (SEM) for each group. Statistical analysis was performed using the ‘Two‐way ANOVA analysis of variance (ANOVA) followed by *Bonferroni's multiple comparisons* test and ‘Unpaired t‐test’ followed by Welch's correction. The data were analyzed using Graph pad Prism 8 software (California, USA). The value of *p* < 0.05 was kept as the level of significance in the expressed data (Table 1).

**TABLE 1:** Particle size and zeta potential measurement.

Sample name	Particle size (d.nm)	Zeta potential (mV)	Polydispersity index (PI)
Plasma fraction (E5)	411 ± 3.39	−10 ± 0.42	0.53 ± 0.01

## 6 RESULTS

### 6.1 TEM

**FIGURE 1:**
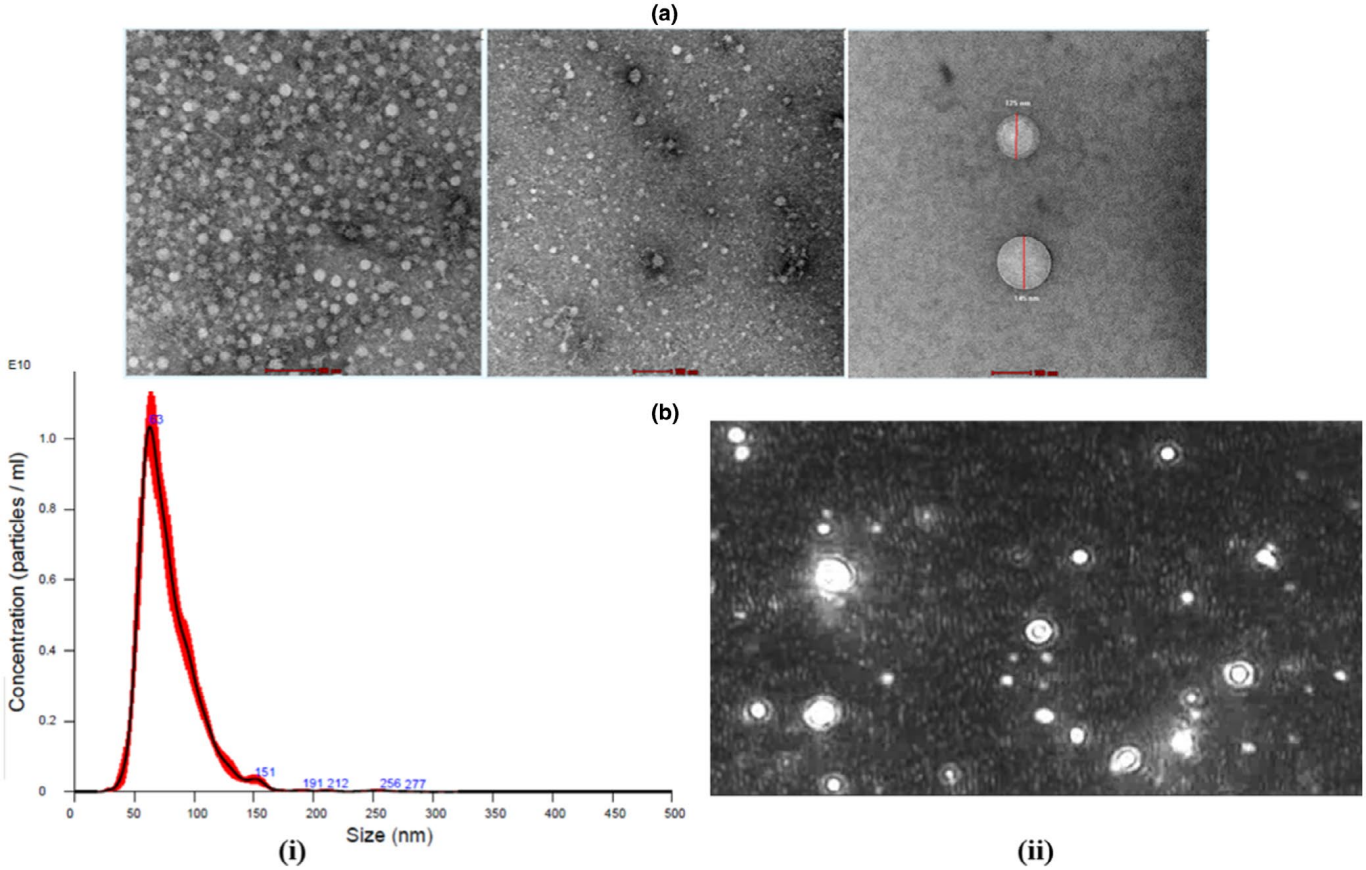
(a) Morphology of plasma‐derived exosome observed by TEM. Scale bar = 100 nm (b) Representative NTA distribution profiles of exosomes derived from plasma including (i) size distribution plot and (ii) screen shot of the corresponding video.

The morphology and size of plasma‐derived exosome was studied using TEM. The size of the exosome was observed between 50 and 150 nm ranges as shown in Figure 1a.

### 6.2 Particle size and zeta potential

Particle size and zeta potential of plasma fraction (E5) were found to be 411 d.nm and − 10 mV respectively.

### 6.3 Nanoparticle tracking analysis

The size and particle concentrations of E5 were found to be 78.4 ± 0.7 d nm and 4.25–1.65 ± 10^10^ particles/ml respectively (Figure 1b). The size and concentrations of nanoparticles was found to be in the range of exosomes recommended particle size range.

### 6.4 Proteomic analysis

**FIGURE 2:**
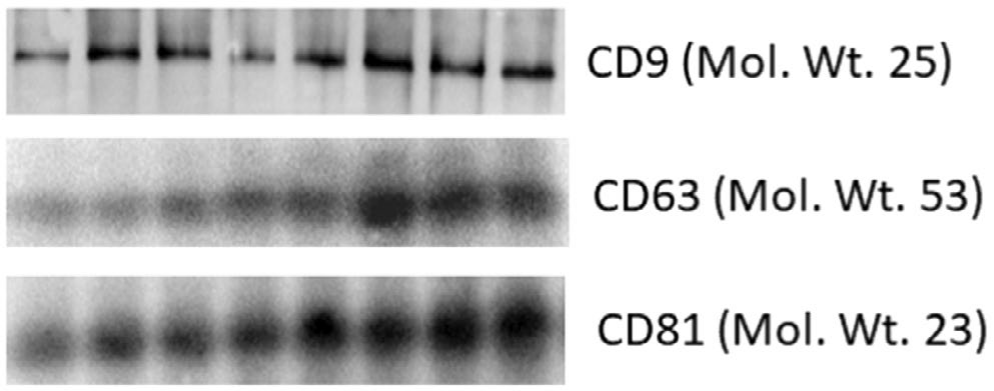
Western blot analysis of CD9, CD63, and CD81 in plasma‐derived exosome samples.

To understand the changes from porcine plasma to E5 fraction at the proteome level, proteomic analysis was carried out using O‐HRLCMS. A combined total of 3078 proteins were identified in the samples. The data obtained from analysis showed the change in the proteins mass peaks as compared to plasma. At the proteome level, remarkable changes were observed between porcine plasma and the isolated E5 fraction (Figure 2 a, b in File S1). A majority of the proteins have been removed with E5 preparation and a few protein fractions have been enriched in the E5 sample which have potential ability in the anti‐aging efficacy. Few fractions were found to be dominant as compared to plasma proteins which could be targeted as biomarkers in future studies.

### 6.5 Western blotting

Western blot analysis confirmed the expression of exosome markers CD9, CD63, and CD81 in the plasma‐derived exosome samples (Figure 2).

### 6.6 Effect of E5 on body weight

**FIGURE 3:**
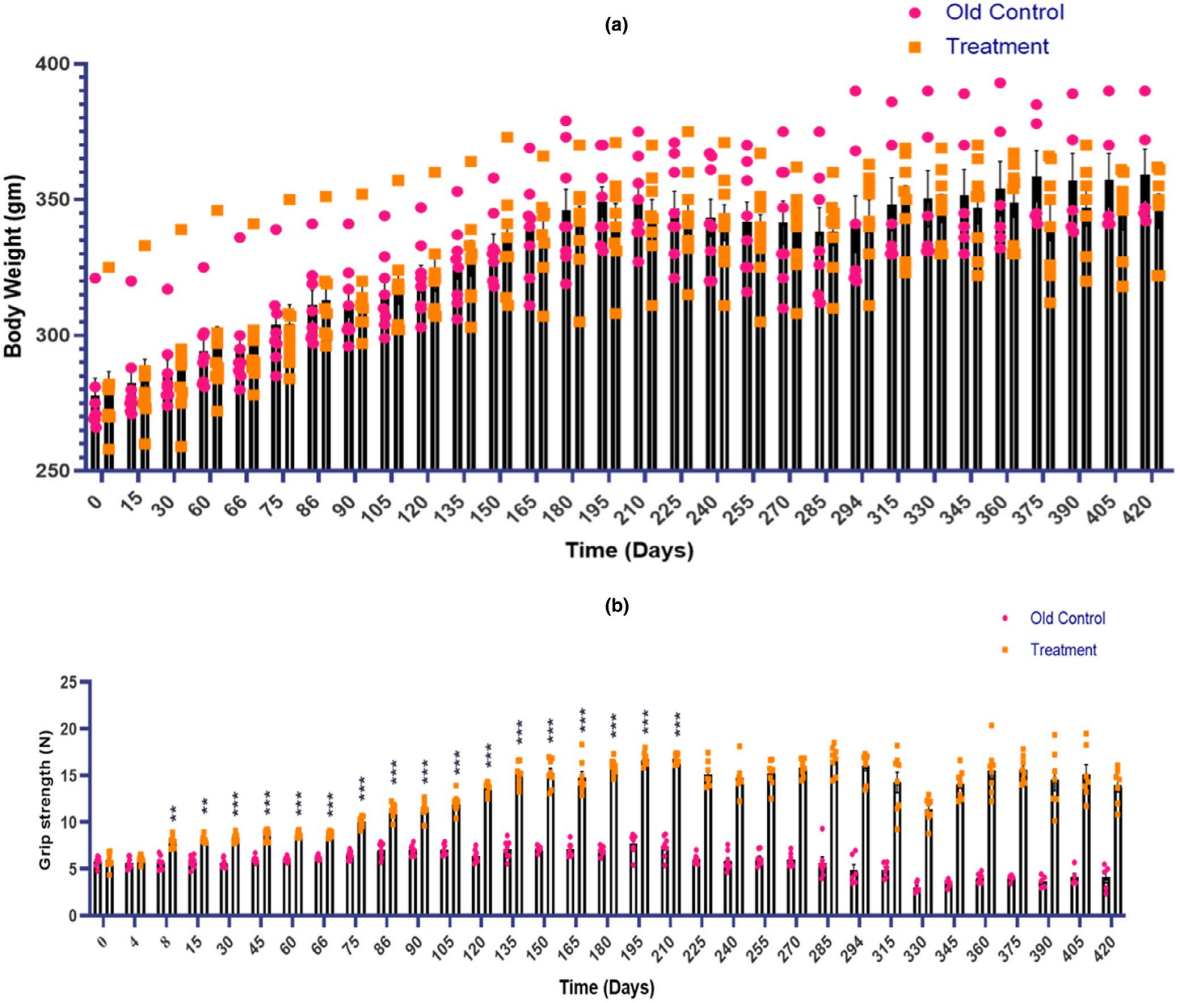
Effect of E5 treatment on (a) Body weight and (b) Grip strength. Data were expressed as mean ± SEM. Statistical analysis by ‘Two‐way ANOVA’ followed by *Bonferroni's multiple comparison* test. ***p < 0.001 and **p < 0.01 as compared to the old control group. (*p* < 0.05 was considered statistically significant).

The E5‐treated group of animals did not show any significant change in body weight when compared with the old control group (Figure 3a).

### 6.7 Effect of E5 on grip strength

The grip strength test is used to measure the neuromuscular function as maximal muscle strength of forelimbs and combined forelimbs and hind limbs. E5 treated group of animals showed significant improvement in grip strength when compared with the old control group of animals (***p < 0.001 and **p < 0.01). After 150 days of treatment, there was at least a 2–3‐fold increase in muscle strength of the treated group (Figure 3b).

### 6.8 Effect of E5 on biochemical parameters

**FIGURE 4:**
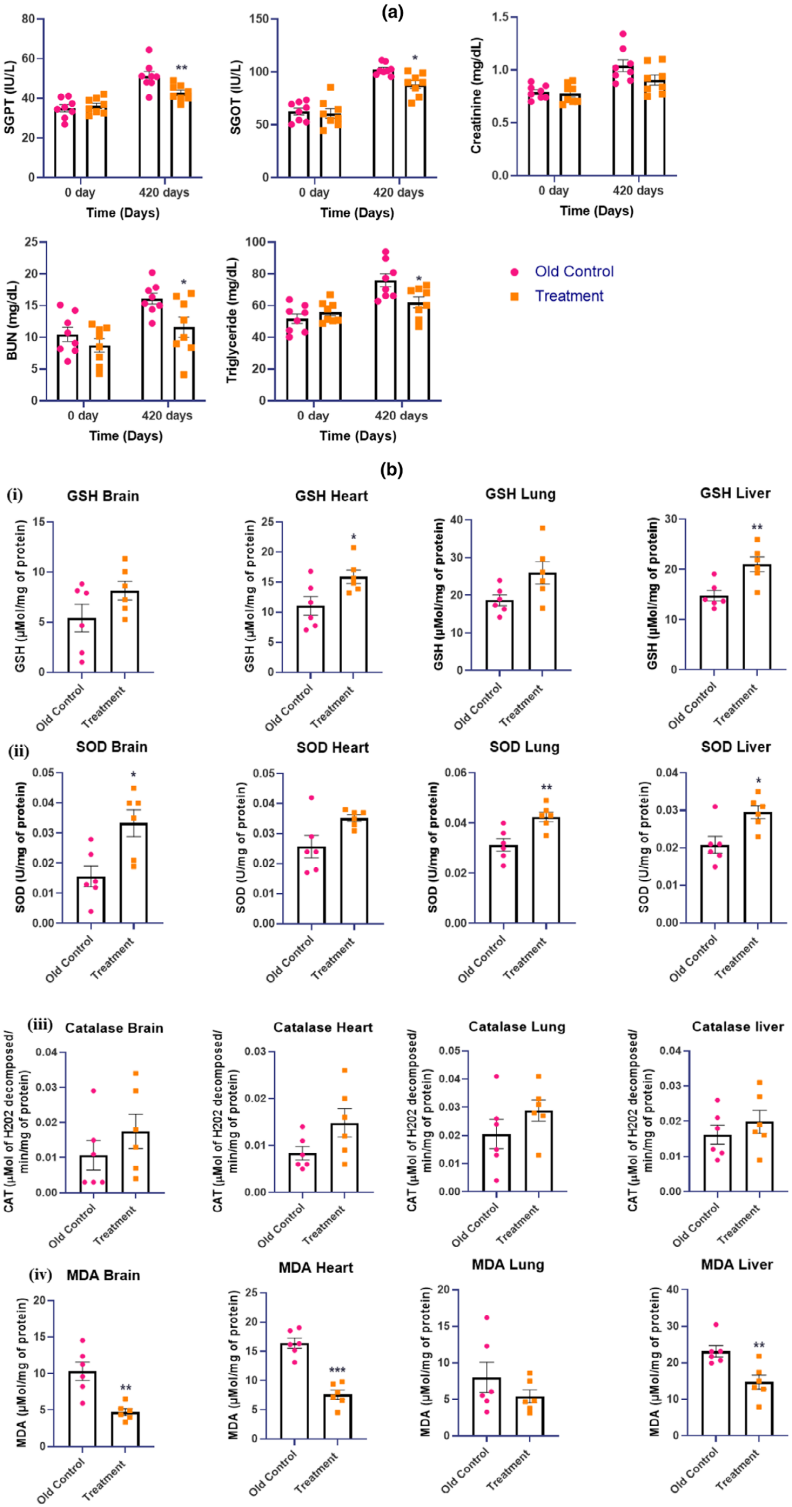
Effect of E5 treatment on (a) Biochemical parameters (SGPT, SGOT, Creatinine, Bun, Triglyceride). Data were expressed as mean ± SEM. Statistical analysis by ‘Two‐way ANOVA (Analysis of variance)’ followed by *Bonferroni's multiple comparison* test. **p < 0.01 and *p < 0.05 as compared to the old control group. (*p* < 0.05 was considered as statistically significant). (b) Oxidative stress parameters such as (i) GSH (ii) SOD (iii) Catalase (iv) MDA on day 420. Data were expressed as mean ± SEM. Statistical analysis by ‘Unpaired t‐test’ followed by Welch's correction. *p < 0.05, **p < 0.01, and ***p < 0.001 as compared to the old control group. (*p* < 0.05 is considered statistically significant).

Serum glutamate pyruvate transaminase (SGPT) concentration was significantly decreased in the E5‐treated group when compared with the old control group of animals (**p < 0.01). The E5‐treated group of animals showed a significant reduction in SGOT concentration when compared with the old control group (*p < 0.05). The kidney function test parameters like creatinine and BUN concentration were determined in the E5‐treated and old control group of animals. The E5‐treated group of animals did not show any significant difference in creatinine concentration when compared with the old control group. BUN was significantly decreased in the E5‐treated group of animals when compared with the old control group (*p < 0.05). The E5‐treated group showed a significant reduction in TG concentration when compared with the old control group (*p < 0.05) (Figure 4a).

### 6.9 Effect of E5 on oxidative stress parameters

The level of GSH, SOD, Catalase, and MDA (Figure 4b) were determined in the brain, heart, lung, and liver tissue homogenate. E5 treated group of animals showed a significant increase in GSH levels in heart and liver tissue homogenates (*p < 0.05 and **p < 0.01). SOD level was significantly increased in the brain, lung, and liver tissue homogenate of the E5 treated group when compared with the old control group (*p < 0.05 and **p < 0.01 respectively). E5 treated group did not show any significant difference in catalase concentration of brain, heart, lung, and liver tissue homogenate when compared with the old control group.

E5 treated group of animals showed a significant reduction in the MDA concentration of brain, heart, and liver tissue homogenates when compared with the old control group (**p < 0.01, and ***p < 0.001, and **p) (Figure 4b).

### 6.10 Effect of E5 on inflammatory cytokines

#### 6.10.1 Tumour necrosis factor‐alpha (TNF‐α)

**FIGURE 5:**
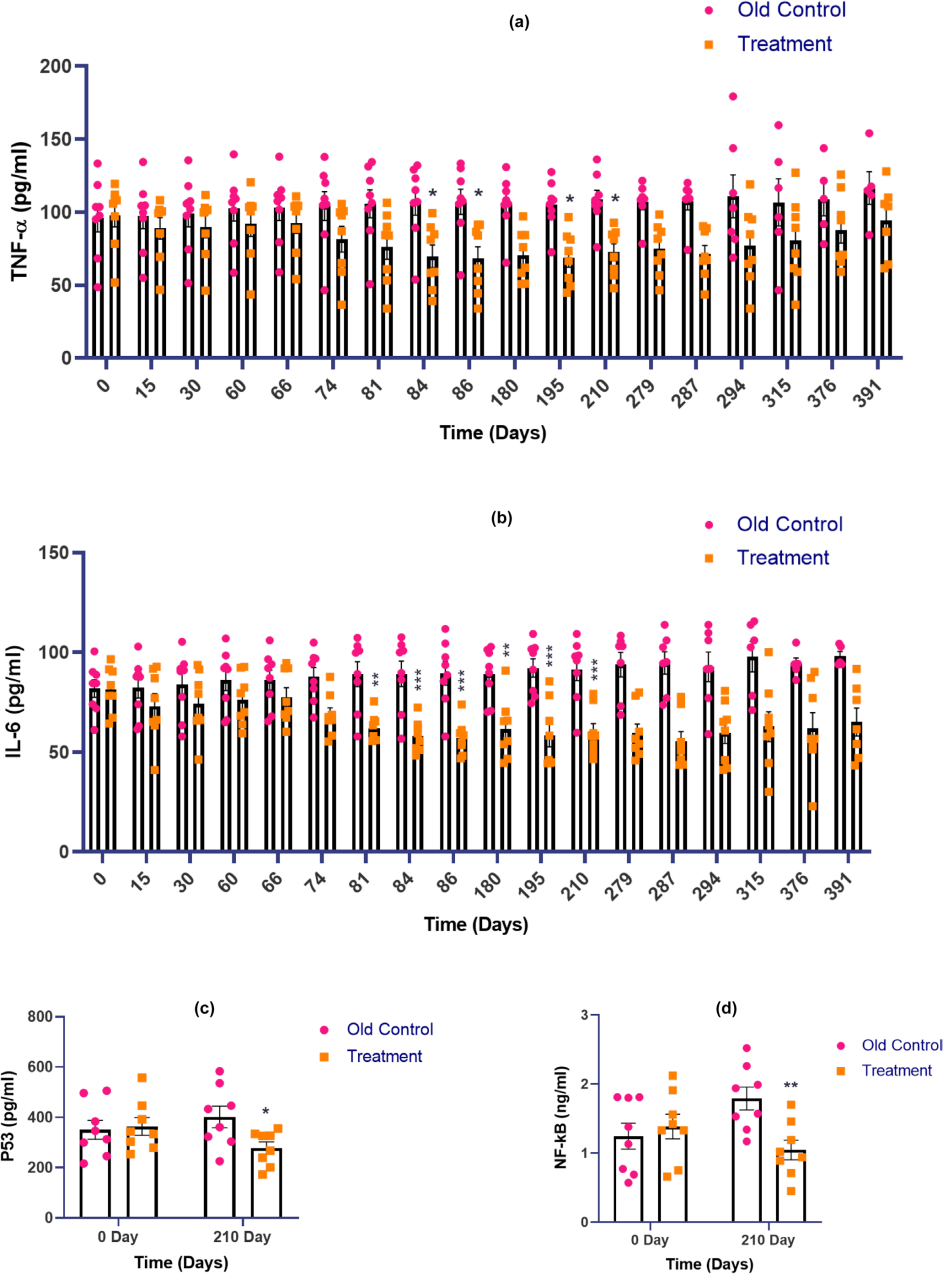
Effect of E5 on (a) TNF‐α (b) IL‐6 (c) p53 and (d) NF‐κB concentration. Data were expressed as mean ± SEM. Statistical analysis by ‘Two‐way ANOVA’ followed by *Bonferroni's multiple comparison* test. *p < 0.05, **p < 0.01 and ***p < 0.001 as compared to the old control group (*p* < 0.05 was considered as statistically significant).

TNF‐α concentrations were reduced after the third dose of E5 on the 84, 86, 195, and 210th day of the study. E5 treated group of animals showed a significant reduction in TNF‐α concentration when compared with the old control group of animals. (*p < 0.05) (Figure 5a).

#### 6.10.2 Interleukin‐6 (IL‐6)

The E5‐treated group of animals showed a significant reduction in IL‐6 concentration when compared with the old control group of animals. A significant reduction was observed in IL‐6 concentration of E5 treated group of animals after the second dose of E5 on the 81, 84, 86, 180, 195, 210, 279, 287, 294, 315, 376, and 391 days of the study (***p < 0.001 and **p < 0.01) (Figure 5b).

#### 6.10.3 Effect of E5 on p53 concentration

The p53 transcription factor pathway plays a pivotal role in regulating apoptosis and senescence, rendering it a compelling candidate for the development of senolytic drugs. The E5‐treated group of animals showed a significant decrease in p53 concentration on the 210th day of the treatment when compared with the old control group of animals (*p < 0.05) (Figure 5c).

#### 6.10.4 Effect of E5 on NF‐κB concentration

Transcriptional activity of NF‐κB is increased in a variety of tissues with aging and is associated with numerous age‐related degenerative diseases. E5 treated group of animals showed a significant reduction in NF‐κB concentration when compared with the old control group of animals (**p < 0.01) (Figure 5d).

#### 6.10.5 Effect of E5 on clinical observation and necropsy

In the last stages of aging, body weight frequently declines in rats and eventually results in death. In the initial days of the study, the mobility of the rats was normal in both groups. In the last days of the study, the animal had some ataxia and splay causing problems with movement. There was no sign of tumors or abnormal growth in both groups. Skin and coat were dull and dirty. Particularly on the backs of the rats in both groups, the debris appeared as a waxy, yellowed coating around the hair follicles. Because older rats may not be able to reach their upper back to clean, this area may show signs of aging first. The tails of both groups of animals showed faecal staining and accumulation around the perineum, inflammation, and discoloration.

The Necropsy of both group animals showed normal structure and architecture. While in the old control and treatment group, some animal's livers showed fatty liver, enlargement of the heart, and splenomegaly was observed. Necrotic lesions were observed in the lungs and kidneys of some animals (Necropsy report in File S1).

#### 6.10.6 Effect of E5 on the survival rate of rats

The survival was compared between old control and E5 treated groups. No deaths occurred up to 33 months of age in either of the groups that is, old control and treatment groups. The first death occurred in the old control group at an age of 33.99 months thereafter, mortality was seen in the old control group at the age of 35 and 37.89 months. The maximum age of the old control group animal was 39.49 months as shown in Table 2. Remarkably, in the E5 treated group, the first death occurred at the age of 38 months. The Mortality among the other animals in the E5‐treated group was noted between 39 and 41 months of age. The E5‐treated group of animal's average longevity increased to 40.67 months compared to control group which was 37.08 months. The increase in survival was observed in E5 treated group, thereby signifying increase in the life‐span of animals.

**TABLE 2:** Lifespan detail of old control and E5‐treatment group of animals.

Old control	Average lifespan (months)	Treatment	Average lifespan (months)
Animal #		Animal #	
C1	35.00	T1	38.00
C2	33.99	T2	39.23
C3	35.00	T3	38.59
C4	37.89	T4	40.07
C5	38.03	T5	39.00
C6	39.49	T6	48.07
C7	39.26	T7	40.92
C8	38.00	T8	41.49
Average lifespan in months	37.08		40.67

One of the rats (named ‘Sima’) by surviving 48 months of age created a world record (Table 2). She received the Diamond Longevity award for longest living rat from the North of England Rat Society and the news was covered in “The Guardian” newspaper (Katcher 2023), (shared as File S1a).

**FIGURE 6:**
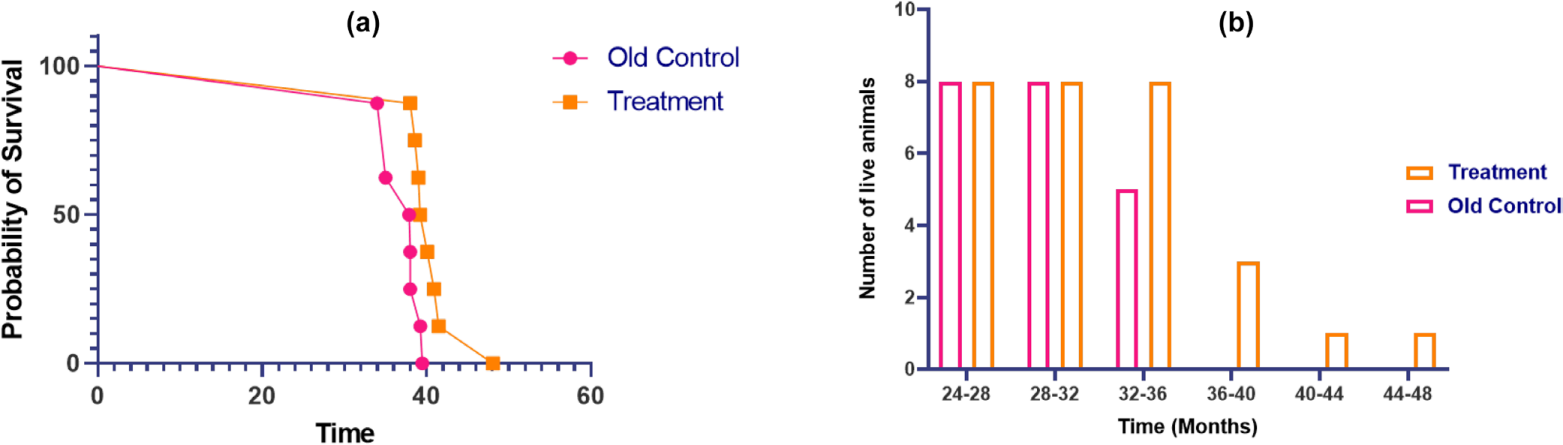
(a) Survival curve (b) Survival data of E5‐treated and old control groups of rats.

As shown in Figure 6a,b, at the end of 40 months, zero and three animals survived in the old control and E5 treated groups, respectively. The mean survival age of old control group was found to be 37.08 months and that of E5 treated group was 40.67 months. A comparable increase in the survival rate of animals was observed in the E5 treated group.

#### 6.10.7 Effect of E5 on histopathology of vital organs

Haematoxylin and eosin stains were used to investigate the effect of E5 on the vital organs of rats. Kidneys and liver tissue of the old control rat showed oedema and mild neutrophil infiltrations while the E5 treatment group showed normal structure and architecture. The brains of old control rats showed distended structure, mild atrophic structure, mild multifocal vacuolar changes, and scattered neuronal degeneration as compared to the E5 treatment group. The ovaries, lungs, and uterus of the old control rat showed mild inflammatory cell infiltrations and structural deformities, while the treatment group of rats showed normal structure and architecture of the ovaries, lungs, uterus, and spleen (Figure 3 in File S1).

## 7 DISCUSSION

This is a unique study wherein we have used 24‐month‐old female rats and monitored them from the age of 24 months until the end of their lifespan with and without E5 treatment. Aging is a complicated occurrence marked by a steady loss in physiological functioning and a rise in disease vulnerability (Hamezah et al., 2017). The age‐associated decline in muscle function and motor coordination was assessed by testing grip strength. It was interesting to note that, the grip strength of animals treated with E5 was significantly improved as compared to old control rats. The E5 improves muscle coordination and functions, by 2–3‐fold as measured by a grip strength meter. Also, we observed that, in comparison to the older control rats, treatment with the E5 did not reveal any alteration in the body weight of animals.

Oxidative stress and inflammation are two fundamental physiological processes underlying many different types of pathologies. Oxidative stress results from over‐production of reactive oxygen species in cells. ROS has gained significant prominence among the different ideas that seek to explain the aging process (Vina et al., 2013). This may occur due to excessive production of ROS or decrease in capacity to eliminate or neutralize ROS. At higher concentrations, ROS can interact with biomolecules and impair their functioning. Measuring the levels of MDA which is the end‐product of polyunsaturated fatty acid peroxidation, reveals the levels of cellular ROS. As anti‐oxidants play a crucial role in ROS defense mechanism, we studied the effect of E5 treatment on the levels of anti‐oxidants and MDA. E5 treatment had great potential to improve the levels of SOD, catalase, GSH and the level of MDA was reduced to a great extent as compared to the old control rats. Therefore, we conclude that the E5 treatment showed a strong anti‐oxidant defense mechanism in response to the formed oxidative stress in aging animals.

Inflammation has been increasingly recognized as an important pathophysiological phenomenon in aging. An increased concentration of inflammatory cytokines is thought to play a critical role in immune response remodelling in old age, with evidence leading to a failure to fine‐control systemic inflammation as a sign of failed aging (Rea et al., 2018). This remodelling of cytokine expression patterns, with a gradual inclination towards a pro‐inflammatory phenotype, is referred to as “inflammaging.” Despite many studies, there is still no clear understanding of the origin of “inflammaging,” which underpins the majority of age‐related disorders such as atherosclerosis, diabetes, Alzheimer's disease, rheumatoid arthritis, cancer, and aging itself (Rea et al., 2018). Plasma derived exosome fraction (E5) treatment exhibited great potential in reduction of pro‐inflammatory cytokines like TNF‐α and IL‐6 markers of chronic inflammation.

The NF‐κB signalling pathway is one of the most well‐studied pathways associated with inflammation, and its implications in the aging process have garnered significant interest. This pathway, governed by the transcription factor NF‐κB, plays a pivotal role in regulating genes connected to inflammation. During the aging process, our bodies accumulate senescent cells. Importantly, the activation of NF‐κB signalling promotes cell senescence and has been shown to reduce the lifespan of fruit flies and mice (Moskalev et al., 2011; Rovillain et al., 2011). In this study, we observed that the E5 treatment significantly reduced the levels of released cytokines and NF‐κB concentration in rat blood plasma as evidence of its potent anti‐inflammatory action.

Aging is strongly correlated with changes in DNA methylation. DNA methylation and epigenetic alterations have been directly linked to longevity in wide array of organisms. The most important methyltransferase in DNA methylation is DNMT1, which also has the same function of regulating cell cycle as p53. Our group has already published DNA methylation study linked with p53. (Haghani et al., 2023; Lu et al., 2021, 2023). Aging has been identified as ‘epigenetic drift’ by Jean Pierre Issa (Issa, 2014) and many other authors after that. Our previous study measured on Horvath methylation clock changes in the epigenetic age of treated rats thereby demonstrating that this drift is somewhat mitigated by E5 treatment (Horvath et al., 2024). Regarding potential role of exosomes: One of the major changes identified in an aging cell is the gradual loss of longer transcripts (Stoeger et al., 2022) and the authors observe that longer transcripts are associated with genes identified for longevity. We have confirmed through an agarose gel study that E5 contains longer transcripts. Replacing the missing transcripts can be speculated one of the potential mechanism of actions of the exosomes from our treatment.

p53 plays a central role in responding to DNA damage and determines the outcome of the DNA damage checkpoint response by regulating cell cycle arrest and apoptosis. However, persistent activation can result in cell death and organismal aging (Vaddavalli & Schumacher, 2022). p53 acts as a transcription factor with critical roles in regulating the cell cycle, DNA repair, apoptosis, and responses to cellular stress. In addition to triggering cell growth arrest and apoptosis, the activation of p53 also exerts influence over cellular senescence and the aging process (Rufini et al., 2013). In old rats, E5 treatment showed a significant reduction in the concentration of p53 in rat plasma, ultimately protecting the cells against apoptosis and DNA damage.

The enzymes and the biochemical regulators in the liver play an important role in age‐related disease conditions (Tan et al., 2018). Hence, to study the impact of E5 treatment on vital organs, we estimated the levels of SGPT and SGOT to monitor liver function, the levels of BUN and creatinine to monitor kidney function, and TGs concentration to monitor risk of atherosclerosis, heart disease and liver function. We found that the levels of all these biochemical markers were significantly attenuated by the E5 when compared with the old control group of animals. E5 may regulate the levels of these biochemical markers by improving the functions of organ systems.

Histological examination in the present work indicates age‐related changes in old control rat tissues as compared to the E5‐treated rats. E5‐treated rats showed remarkable difference in multifocal vacuolar change with scattered neuronal degeneration in the brain as compared with old control rats. Additionally, the lungs displayed emphysematous patches, and other vital organs exhibited mild tissue damage in comparison to the control rats.

The mechanism behind E5's effects is intriguing. The cellular secretome, which E5 taps into, changes as mammals get older (Stoeger et al., 2022). The study revealed a significant impact of E5 treatment on the longevity of rats, exemplified by Sima, who lived up to an astonishing 48 months as compared to the old control group with the maximum age reaching 39.49 months. Notably, the E5 treatment group exhibited delayed mortality. Sima's notable longevity highlights the potential of E5 treatment to prolong the lifespan of rats, leading to acknowledgement by the North of England Rat Society and publication of article in “The Guardian.”

In the past few years, a significant amount of research has been devoted to investigating aging‐related concerns. Aging is associated with physical disability and distinguished by cellular and organismal alterations such as oxidative stress, chromosomal erosion, and endoplasmic reticulum stress which results in diminished tissue functioning (Fried et al., 2001; López‐Otín et al., 2016). Changes in intercellular communication are one of the characteristics linked with aging (Franceschi & Campisi, 2014; Zhang et al., 2013). Several agents have been demonstrated to extend lifespan in various species, as well as delay or reverse a multitude of age‐related characteristics in mice, such as cognitive decline, cardiac problems, and cancer (Guo et al., 2022; Pappas & Nagy, 2019). Young blood plasma has been recognized for its beneficial effects on various organs in mice and rats, a recent research study presented compelling evidence supporting the epigenetic age reversal in rats through the administration of exosomes containing plasma fractions E5 obtained from young adult pigs. Porcine plasma‐derived exosomes were used in the current experiment; these therapies are efficient at improving cognitive performance in the aged population (Horvath et al., 2024).

According to this research, plasma derived E5 treatment may be important player in a number of age‐related disorders. Based on the study findings, we infer that the treatment of plasma‐derived exosome increases antioxidant markers, reduces concentrations of cytokines, NF‐κB, and p53, improves the vital organs integrity and ultimately it increases both lifespan as well as health‐span of rats. This outcome is worth investigating further in order to explore potential applications of E5 in the field of aging research.

Our findings suggested that young porcine derived exosomes has the ability to reverse the aging process and it leads to overall extension in the lifespan and healthspan of aging animals. We would like to explore the potential of using “young” plasma‐derived exosomes versus ‘old’ plasma‐derived exosomes, which will be an exciting area of research in the context of anti‐aging therapy. The future studies will likely focus on understanding the precise mechanism by which exosomes offer anti‐aging effect at the cellular or systemic level. The preclinical trials exploring the efficacy of E5 in treating age‐related diseases like Alzheimer's, Parkinson's, and osteoporosis may be conducted in the future.

## 8 CONCLUSION

It is noteworthy that plasma fraction treatment is consistently found to be effective in extending both, lifespan and health‐span of SD rats. Our study indicates that certain tissues can be rejuvenated by administering plasma between species, specifically between pigs and rats. Such outcomes offer insights into shared biological mechanisms across species, which are potentially relevant to human health. Further research is required to determine the identity of the effective rejuvenating components present in the plasma‐derived fraction.

## AUTHOR CONTRIBUTIONS

Kavita Singh, designed the studies/protocols, secured necessary study approvals, critically monitored in‐house as well as collaborative project work and provided technical guidance in capacity of Principal investigator. Shraddha I. Khairnar, Tanuja T. Yadav, Neha Gupta and Jay Arora conducted experiments, collaborated on experiment design, performed data analysis and contributed to manuscript drafting. Harold L. Katcher supervised in development of hypothesis, study design and data analysis. Akshay Sanghavi supervised the overall direction of the project, provided critical in‐puts and secured financial support. All authors participated in manuscript editing and offered critical feedback that significantly influenced the research, analysis and manuscript development.

## CONFLICT OF INTEREST STATEMENT

We declare no conflict of interest.

## FUNDING INFORMATION

NGO Heales (Healthy Life Extension Society, Belgium).

## Supporting information


File S1.

File S1a.

## Data Availability

Data will be available on reasonable request to the corresponding author.
