# Supplementary material for: E5 treatment showing improved health‐span and lifespan in old Sprague Dawley rats
Source: Aging Cell. 2024 Sep 19;23(12):e14335. doi: 10.1111/acel.14335 (PMC11634717; doi:10.1111/acel.14335)
Supplement: Supplementary file 1 — File S1. [file ACEL-23-e14335-s001.docx]

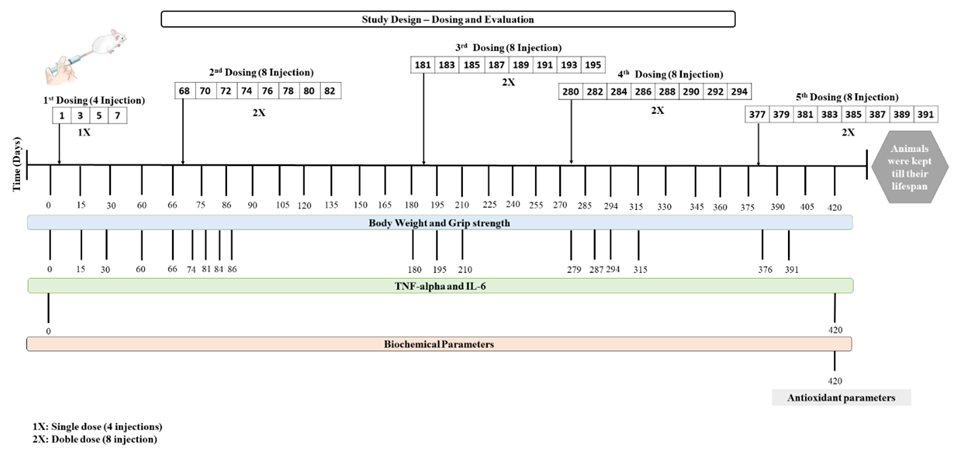
**Figure 1. Study design and dosing schedule**


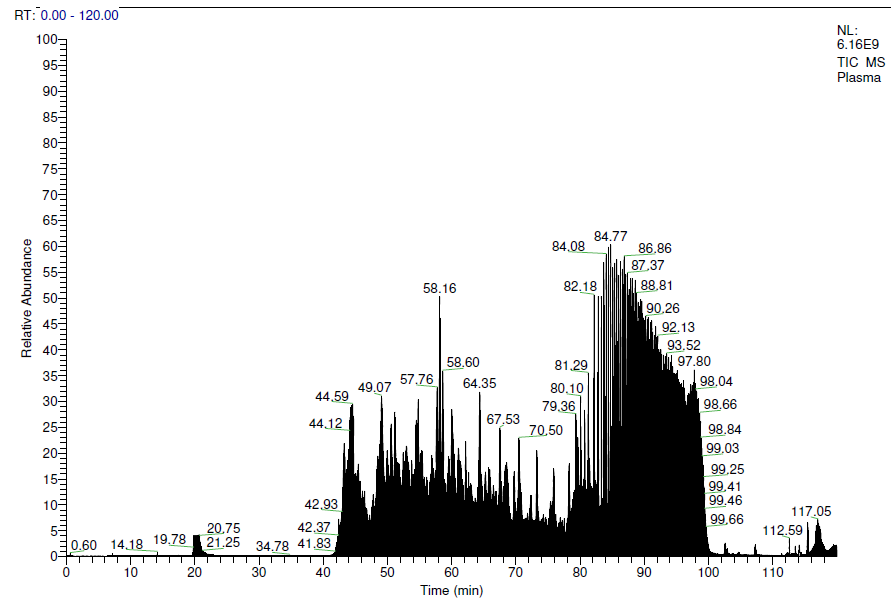


**
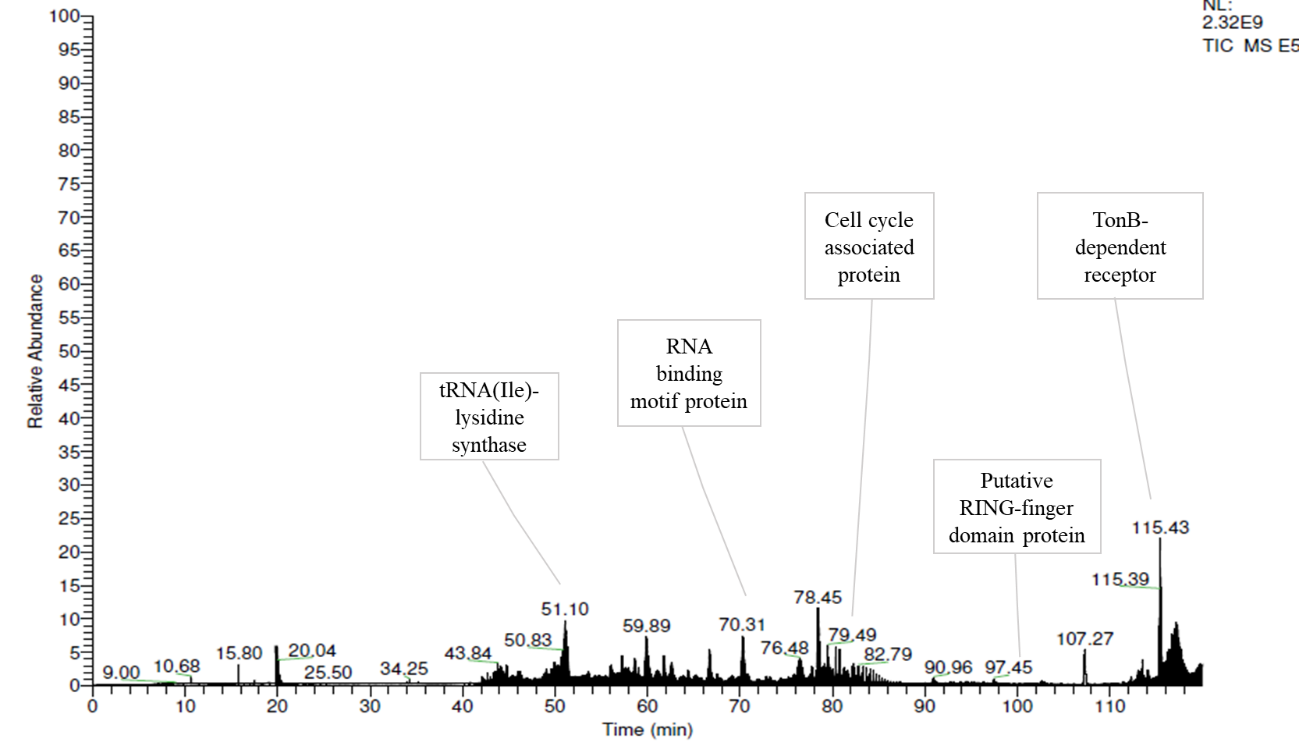
**

**Figure 2. Mass spectra of (A)** **porcine plasma and (B) E5 fraction.**

**
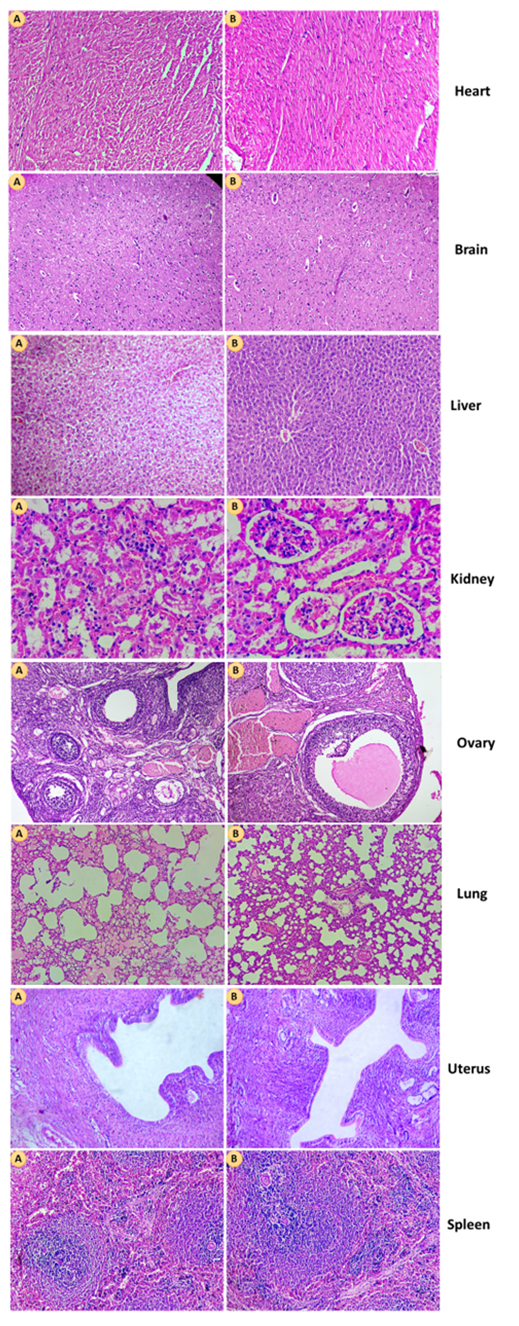
**

**Figure 3. Histopathological evaluation of vital organs after the treatment with E5**

**(**A: Old control and B: Treatment).

**Table 1. Necropsy report of an old control group of animals**

| **Rat No.** | **Brain** | **Heart** | **Lung** | **Liver** | **Spleen** | **Kidney** | **Small intestine** | **Large intestine** | **Bladder** | **Ovary & Uterus** |
| --- | --- | --- | --- | --- | --- | --- | --- | --- | --- | --- |
| 1 | Normal | Normal | **Necrotic** | Normal | Normal | Normal | Normal | Normal | Normal | Normal |
| 2 | Normal | **Enlarged** | Normal | **Fatty** | Normal | Normal | Normal | Normal | Normal | Normal |
| 3 | Normal | Normal | Normal | Normal | Normal | Normal | Normal | Normal | Normal | Normal |
| 4 | Normal | **Enlarged** | Normal | **Fatty** | **Enlarged** | Normal | Normal | Normal | Normal | Normal |
| 5 | Normal | **Enlarged** | **Necrotic** | **Fatty** | Normal | Normal | Normal | Normal | Normal | Normal |
| 6 | Normal | Normal | Normal | Normal | **Enlarged** | **Necrotic** | Normal | Normal | Normal | Normal |
| 7 | Normal | Normal | Normal | **Fatty** | Normal | **Necrotic** | Normal | Normal | Normal | Normal |
| 8 | Normal | Normal | Normal | Normal | Normal | Normal | Normal | Normal | Normal | Normal |

**Table 2. Necropsy report of an E5-treated group of animals**

| **Rat No.** | **Brain** | **Heart** | **Lung** | **Liver** | **Spleen** | **Kidney** | **Small intestine** | **Large intestine** | **Bladder** | **Ovary & Uterus** |
| --- | --- | --- | --- | --- | --- | --- | --- | --- | --- | --- |
| 1 | Normal | Normal | Normal | Normal | Normal | Normal | Normal | Normal | Normal | Normal |
| 2 | Normal | Normal | Normal | Normal | Normal | Normal | Normal | Normal | Normal | Normal |
| 3 | Normal | Normal | Normal | Normal | Normal | Normal | Normal | Normal | Normal | Normal |
| 4 | Normal | **Enlarged** | **Necrotic** | **Fatty** | Normal | Normal | Normal | Normal | Normal | Normal |
| 5 | Normal | Normal | Normal | **Fatty** | Normal | Normal | Normal | Normal | Normal | Normal |
| 6 | Normal | **Enlarged** | Normal | Normal | Normal | Normal | Normal | Normal | Normal | Normal |
| 7 | Normal | Normal | Normal | Normal | **Enlarged** | Normal | Normal | Normal | Normal | Normal |
| 8 | Normal | Normal | Normal | Normal | Normal | Normal | Normal | Normal | Normal | Normal |

**Table 3. Lifespan detail of old control group of animals**

| **Old Control** | **(mm/dd/yy)** | | **Lifespan (Months and days)** | **Average Lifespan (Months)** |
| --- | --- | --- | --- | --- |
| **Animal #** | **Birth** | **Death** |  |  |
| C1 | 12-2-2018 | 11-2-2021 | 35 months | 35.00 |
| C2 | 2-1-2019 | 12-1-2021 | 33 months, 30 days | 33.99 |
| C3 | 3-4-2019 | 2-4-2022 | 35 months | 35.00 |
| C4 | 3-4-2019 | 5-1-2022 | 37 months, 27 days | 37.89 |
| C5 | 3-4-2019 | 5-5-2022 | 38 months, 1 day | 38.03 |
| C6 | 2-4-2019 | 5-19-2022 | 39 months, 15 days | 39.49 |
| C7 | 3-4-2019 | 6-12-2022 | 39 months, 8 days | 39.26 |
| C8 | 4-15-2019 | 6-15-2022 | 38 months | 38.00 |
| Average Lifespan in Months | | | | 37.08 |

**Table 4. Lifespan detail of an E5-treated group of animals**

| **Treated** | **(mm/dd/yy)** | | | **Lifespan (Months and days)** | **Average Lifespan (Months)** |
| --- | --- | --- | --- | --- | --- |
| **Animal #** | **1st injection** | **Birth** | **Average Lifespan (Months)** |  |  |
| T1 | 1-30-2021 | 12-15-2018 | 2-15-2022 | 38 months | 38.00 |
| T2 | 1-30-2021 | 1-18-2019 | 4-25-2022 | 39 months, 7 days | 39.23 |
| T3 | 1-30-2021 | 2-18-2019 | 5-6-2022 | 38 months, 18 days | 38.59 |
| T4 | 1-30-2021 | 1-18-2019 | 5-20-2022 | 40 months, 2 days | 40.07 |
| T5 | 1-30-2021 | 2-24-2019 | 5-24-2022 | 39 months | 39.00 |
| T6 | 1-30-2021 | 2-28-2019 | 03-03-2023 | 48 months, 2 days | 48.07 |
| T7 | 1-30-2021 | 2-28-2019 | 7-26-2022 | 40 months, 28 days | 40.92 |
| T8 | 1-30-2021 | 3-30-2019 | 9-14-2022 | 41 months, 15 days | 41.49 |
| Average Lifespan in Months | | | | | 40.67 |

**Table 5. Histopathological observation of old control group of animals.**

| **Organ** | **Observations of an old control group of animals** |
| --- | --- |
| **Brain** | Mildly multifocal vacuolar changes scattered neuronal degeneration |
| **Heart** | No abnormalities were detected |
| **Lung** | Minimally multifocal minimal degree emphysematous patches |
| **Liver** | Diffuse microvesicular degeneration/ glycogen infiltration of moderate severity |
| **Spleen** | No abnormalities were detected |
| **Kidney** | The tubular epithelium showed granular degeneration of mild severity |
| **Ovary** | Increased percentage of atretic follicles |
| **Uterus** | Atrophic myometrium |

**Table 6. Histopathological observation of an E5-treated group of animals.**

| **Organ** | **Observations of an E5-treated group of animals** |
| --- | --- |
| **Brain** | No abnormalities were detected |
| **Heart** | No abnormalities were detected |
| **Lung** | No abnormalities were detected |
| **Liver** | No abnormalities were detected |
| **Spleen** | No abnormalities were detected |
| **Kidney** | No abnormalities were detected |
| **Ovary** | No abnormalities were detected |
| **Uterus** | No abnormalities were detected |
