# Supplementary figures and images for: E5 treatment showing improved health‐span and lifespan in old Sprague Dawley rats
Source: Aging Cell. 2024 Sep 19;23(12):e14335. doi: 10.1111/acel.14335 (PMC11634717; doi:10.1111/acel.14335)

**Supplementary file 1a**


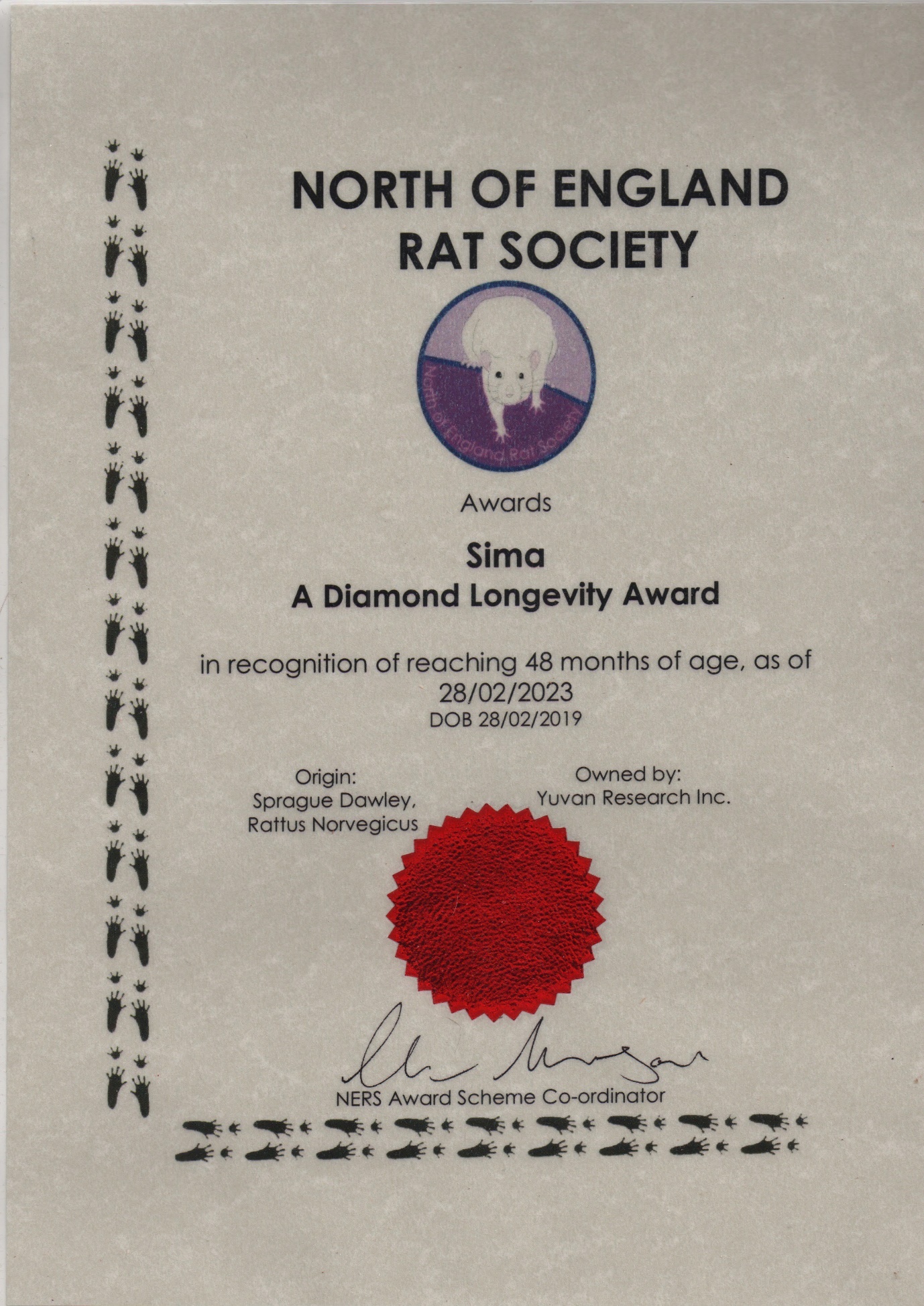

Supplement: Supplementary file 2 — File S1a. [file ACEL-23-e14335-s002.docx]
